# German Review

**Published:** 1891-07

**Authors:** Leonard Pearson


### Selections from Foreign Journals.

### *GERMAN.*

By Leonard Pearson, B. S., V. M. D.

### CANTHARIDES.

Army Veterinarian Tetzner reports, in the June number of the *Zeitschrift für Veterinärkunde*, an experiment in which he studied the action of cantharides in a case of chronic laryngitis in a horse. It is well known that Prof. Liebreich, of Berlin, has recommended the subcutaneous use of a compound of cantharides (cantharidate of potash) in the treatment of Tuberculosis, and that this remedy has been used, with reported good results, in cases of laryngeal tuberculosis and chronic laryngitis in man.

The author had under observation a half-blood saddle mare, 10 years old, that had been affected with a severe cough for two years. The cough was especially violent in dusty places and when the mare was being ridden ; it was so bad at all times as to cause the rider great annoyance, from the fact that the head and neck were carried extended, and constant efforts were made by the mare to relieve herself of the restraint of the bit. Every remedy that promised results had been tried, and had failed to produce, at most, more than a temporary improvement in the symptoms. Even the treatment advised by Prof. Dieckerhoff in this class of cases—intratracheal injections of solutions of tannine, Lugol's solution, and nitrate of silver—was of no avail.

Encouraged by the results that some clinicians had claimed for the Liebreich treatment, Tetzner decided to try the remedy. He administered, in pill form, .25 gm. (gr. iv.) per day, for two days ; then double this dose for two days. No medicine was given for the next two days and then the treatment was recommended where left off. In this it was arranged that periods of treatment and non-treatment, each of two or three days should alternate for some two weeks. Except the first doses, all contained 0.5 grms. (7½ grs.)

During the treatment the urine was examined several times and no changes discovered. The mare's appetite improved, the coat became shiny, and the temperament more lively. At the same time the cough diminished in frequency and intensity until, during a ride of one to two hours, but one or two coughs could be noticed, and these were not of the distressing nature that previously characterized them. This favorable state of affairs lasted some twenty days, after the cessation of the treatment, when the mare commenced to cough again if ridden in the dust. But the improvement is still very marked and encouraging. The author closes by saying, that only future observation can determine if the disease will resume its previous form, but thinks his results should encourage other experiments in the same direction.

---

### ECZEMA.

The following salve is recommended for eczema and parasitic diseases of the skin :

| | | |
|---|---|---|
| ℞ | Acid. salicy. | 5.0 grms. |
| | Creosot, | 2.0 " |
| | Sapo.medicat., | 100.0 " |

M.

*Zeit. f. Veterinärkunde, III., 3.*

---

### LOCAL ANÆSTHESIA.

The following mixture is said to be very effective in producing local anæsthesia :

| | | |
|---|---|---|
| ℞ | Chloroform, | 10.0 grms. |
| | Ether, | 15.0 " |
| | Menthol, | 1.0 " |

M.

The skin is to be moistened with this fluid and kept moist, for one minute before the operation.

*Ibid.*

---

About 100,000 lbs. of bacon which was said to be from Holland, but which was supposed by the authorities to be from America, has recently been seized in Cologne and Diesseldorf, and veterinarians engaged in meat inspection, in various parts of Germany, have been called upon to give their opinion as to the origin of this pork. That the meat came from Holland is undoubted, but the authorities claim that it was imported into this country from America. In Cologne it has been decided that the bacon is from America and it will be destroyed. The experts claim to be able to diagnose our pork by the following characters: the sides are covered with black hair (in Germany and Holland most of the hogs are white); the meat has a peculiar odor of turpentine which is especially noticeable in the cooking, and this odor clings to the hands and instruments for days; the taste is rancid, the water in which it is cooked foams more than is usual and the meat contracts on the bone more than the native variety does. It is said that five to ten per cent. of these sides contained trichinæ. Samples of pork from America, procured through the German Consul showed the same characters.—*Zeit. f. fleisch u. Milchhyg.*, No. 9, '91.

---

### GARGET.

Prof. Kitt, of Munich, has found a bacillus that is constantly present in the udder in cases of mastitis (parenchymatous inflammation of the udder), and pure cultures of which injected into the milk cistern will cause the disease. The author also made experiments of injecting the bacillus of malignant œdema, oidum lactis, micrococcus tetragenous, and culture of soor into the milk cistern, but these produced no inflammation. The bacillus of blue milk and of chicken cholera produced a catarrhal mastitis. The specific bacillus of mastitis (the mastitis bacillus), when rejected, produced, in every case, a severe purulent, indurative mastitis, and even smearing the cultures about the mouth of the teat was sufficient to cause the disease. The inflammation was always confined to the inoculated quarter, and one attack did not give future immunity. Injections, into the udder, of solutions of creoline and iodine had no curative effect.—*Monatsh. f. prat. Thierheilk. II. Bd., H.* 1.

